# Partial Reconstruction of Uterus Cervix in Rat by Decellularized Human Uterine Cervical Scaffold Combined with Adipose-Derived Stem Cells (ADSCs)

**DOI:** 10.1155/2022/6287435

**Published:** 2022-09-12

**Authors:** Yuqi Li, Chunbo Li, Luopei Guo, Xiaotong Liu, Keqin Hua, Xuyin Zhang

**Affiliations:** Obstetrics and Gynecology Hospital of Fudan University, 128 Shen Yang Road, Shanghai 200090, China

## Abstract

Surgical management for cervical malformation remains as the main therapeutic challenge for gynecologists. A theoretical alternative is to generate a bioengineered uterus cervix, which requires scaffold structure and appropriate cellular constituents. Here, human uterine cervical tissue was decellularized with detergents to produce an acellular scaffold that retained extracellular matrix (ECM), characterized through histochemical studies and DNA assessments. Recellularized scaffold was then established by decellularized scaffold reseeding with adipose-derived stem cells (ADSCs) isolated from rats. We tested these bioengineering samples in a rat model of partial cervical defect and found that recellularized scaffold improved regeneration abilities of the uterine cervix, promoted better vascularization, and achieved positive pregnancy outcomes. These results suggest that decellularized human uterine cervical scaffold combined with ADSCs could be used for uterine cervical regeneration and provide insights into treatments for cervical malformation.

## 1. Introduction

Cervical aplasia is a rare condition with an incidence of approximately 1 : 100,000 births [1, 2]. The cervix can be affected by congenital and acquired pathologies; the former arises from abnormal development of the Mullerian ducts, while the latter can be caused by cancer or trauma. Cervical aplasia represents an impairment of the outflow tract in which patients usually present with pelvic pain or haematometra. However, cervical malformations are very difficult to treat and warrant surgical intervention, often requiring extensive reconstruction to restore normal internal genital anatomy. Hysterectomy used to be the main surgical intervention. As surgical techniques advanced, these methods were gradually changed toward conservative approaches such as “canalization” [3]. However, the construction of the cervix is very important to achieve surgical goals including anatomical reconstruction, menstrual restoration, and fertility promotion. At present, a series of materials have been used to repair the cervical defects, such as amniotic membrane and small intestinal submucosa (SIS) [4–7] with limitations including higher incidence of neovaginal neoplasia, infection, and lack of supporting function.

Recently, bioengineering has shown advantages to organ reconstruction and regeneration; this also applies to cervical aplasia. To date, different biomaterials and cell sources for cervicovaginal deformations have been explored, e.g., synthetic scaffolds as silk [8, 9]. Meanwhile, after recognizing the key role of natural ECM in the body, the decellularized matrix has gained importance as scaffolds. Decellularization (DC) refers to the removal of the original cells of organ/tissue by various methods while preserving ECM components, maintaining the three-dimensional structure, and thus providing a suitable environment for seeded cells. Removal of antigenic contents reduces the possibility of inflammatory reactions and potential immune rejection. In this respect, some commercial xenogeneic acellular matrices such as porcine acellular dermal matrix (ADM) or porcine SIS have been shown viable for cervicovaginal reconstructions, resulting in near normal sexual function [4, 10]. However, their thin layer structure presents a limitation for reconstruction of cervix due to the lack of supporting structure. Some experts think nonspecific ECM used for reconstruction may lead to nonideal environment for tissue regeneration in vivo and subsequently results in limited organ functionality [11, 12]. Thus, acquiring tissue-specific biomaterials from natural sources represents a vital and promising avenue to achieve progress in cervical reconstruction.

Decellularized matrix can provide a suitable environment for recellularization (RC), and the key element of RC is the selection of suitable seeded cell types. Since being first isolated from the adipose tissue in 2001 [13], ADSCs have been a commonly used prorepair MSC in regenerative medicine. Compared to other cell types, ADSCs present numerous advantages: sufficient source, easy isolation, and low immunogenicity. Besides, ADSCs are capable of secreting a vast array of angiogenesis-related cytokines such as vascular endothelial growth factor (VEGF), which improves implant biocompatibility [14]. The above characteristics make ADSCs the most promising cell type in regenerative medicine.

**Figure 1:**
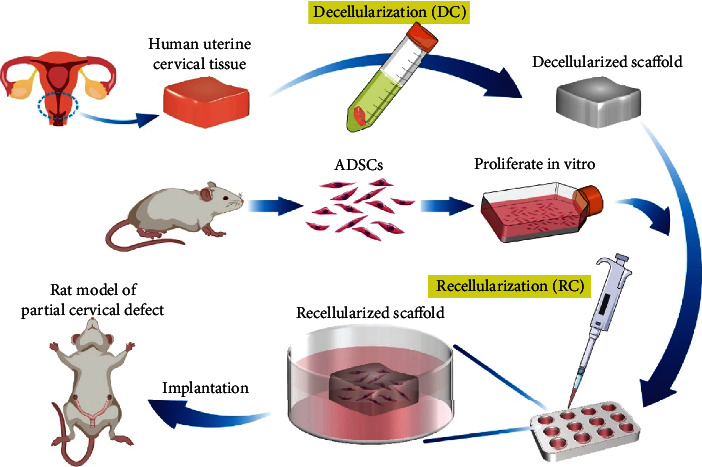
A brief schematic diagram of the experimental design. Human uterine cervical tissue was decellularized with detergents. ADSCs were isolated from rats, cultured, and then reseeded on the decellularized scaffold. After coculture, recellularized scaffold was implanted to a rat model of partial cervical defect.

In the study, we aimed to develop a cervical recellularized scaffold derived from the human cervical decellularized scaffold for uterine cervical regeneration.  Figure 1 depicts the schematic of the experimental design. Firstly, we explored a DC protocol for human uterine cervical tissues and confirmed the removal of cell components and the preservation of ECM. Then, we seeded ADSCs to construct the cell-scaffold compound and demonstrated a better histocompatibility of decellularized cervix with stem cells. Finally, we achieved successful reconstruction of partial cervical defect in rat models and acquired normal functional recovery.

## 2. Materials and Methods

### 2.1. Decellularization of Human Cervical Tissue

Cervical tissue samples were collected from women undergoing hysterectomy for benign uterine diseases or uterine prolapse at the Obstetrics and Gynecology Hospital of Fudan University. Combining multiple detergents with immersion or agitation is a common and convenient option for decellularization [15–17]. In this regard, few studies focus on decellularization of the human uterus, but there have been relevant attempts on animals especially large animal models. Sodium dodecyl sulfate (SDS) is an effective reagent widely used for thicker organs [15, 18, 19]. Zuk et al. described the first successful obtainment of pig whole-uterus scaffolds as a large animal model with two identical perfusion cycles of using SDS and Triton X-100 [13], while sodium deoxycholate (DOC) was considered relatively mild to preserve ECM and once used to human endometrium tissue [20, 21]. Herein, DC was performed and compared in the following two methods:

1. *SDS Method*. Samples were washed in PBS (Servicebio) at 37°C for 1 h, and then underwent two cycles. Each cycle consisted of 4 immerse steps at 37°C in a shaker (100 rpm): 24 h of 0.1% SDS (Sigma-Aldrich), 30 min of ddH_2_O, 30 min of 1% Triton X-100 (Solarbio), and finally 5 h of PBS
2. *DOC Method*. Samples were immersed into 0.25% DOC (Sigma-Aldrich) and 0.25% Triton X-100 solutions at 37°C in a shaker (100 rpm) for 48 h and then washed in PBS for 72 h

The whole performance was conducted under the sterile state. Decellularized scaffolds were stored in PBS containing 1% penicillin/streptomycin (P/S) at 4°C.

### 2.2. Histochemical Studies and DNA Assessments

Decellularized scaffolds and native tissue were fabricated into paraffin-embedded sections. To assess the efficacy of DC and the integrity of ECM, we stained the sections with hematoxylin and eosin (H&E), Masson, and Alcian blue. Furthermore, sections were processed for elastin (Proteintech), collagen IV (Proteintech), and fibronectin (Servicebio). The slides were viewed under an Olympus BX60 Microscope and analyzed by ImageJ.

Sections were stained with 4′,6-diamidino-2-phenylindole (DAPI) solution (Beyotime) and viewed by a fluorescence microscope. For DNA quantification, segments were weighed and homogenized; then, a DNA extraction kit (Tiangen) was used, and measurements of DNA concentration were done on a NanoDrop (Thermo Scientific, USA).

### 2.3. Isolation, Culture, and Identification of ADSCs

Rat ADSCs were obtained with the method of enzyme digestion. Briefly, subcutaneous adipose tissue in the groin area of SD rat was harvested and washed with sterile PBS containing 1% P/S. The tissue was minced and digested with 0.1% type I collagenase (Worthington) at 37°C for 1 hour. After neutralization, the digested mixture was centrifuged, and the pellet was resuspended in rat ADSC growth medium (Cyagen), cultured in 37°C incubator with 5% CO_2_. When it reached 70-80% confluence, cells were subcultured.

APC anti-rat CD29 antibodies (Biolegend), FITC anti-rat CD90 antibodies (Biolegend), PE anti-rat CD34 antibodies (Biolegend), and PerCP/Cyanine5.5 anti-rat CD45 antibodies (Santa Cruz Biotechnology) were used for flow cytometry of ADSCs. The percentages of the cells that reacted to CD markers were analyzed by histogram with FlowJo. To evaluate the potential to differentiate into multilineage cells, the passage three cells were used to osteogenic-, adipogenic-, and chondrogenic-induced differentiation. All mediums were from Cyagen, and the results were verified by the Alizarin Red solution, the Oil Red O, and the Alcian blue solution, respectively.

### 2.4. Recellularized Scaffolds and Scanning Electron Microscopy (SEM)

After being washed with PBS, the decellularized scaffolds were dried by an experimental vacuum freeze dryer (BD Company, USA) and sliced to 5 mm × 10 mm pieces by sterile blades. Samples were exposed to UV radiation overnight under the laminar hood. The total number of 4 × 105 ADSCs was added to each scaffold and incubated in a 12-well culture plate in 37°C incubator with 5% CO_2_. After 4 h, ADSC media were added and changed every 2 days. The coculture of scaffolds and ADSCs was cultured for 1 week and checked daily.

SEM was performed to investigate the microstructure of the decellularized scaffolds and recellularized scaffolds. Samples were fixed by electron microscopy fixative for 2 hours and washed with PBS. Then, they were postfixed by 1% OsO_4_ in PBS, washed, dehydrated by graded ethanol, and dried with a critical point dryer. Finally, the samples were gold sputtered and visualized by a scanning electron microscope.

### 2.5. Implantation of Samples

Mature female Sprague-Dawley rats weighing between 250 and 300 g were obtained from the Shanghai Jiesijie Corporation, and experiments were approved by the Institutional Animal Care and Use Committee of Shanghai Fudan University, China. Rats were randomly assigned to four groups, including recellularized scaffold group, decellularized scaffold group, subtotal excision-only group, and normal control group. After anesthetization, an abdominal midline incision was made to expose the uterus. The cervix was detected by exploring down the uterus, and moist gauze was used to wrap the abdominal viscera to avoid dehydration. A segment of approximately 0.5 cm in length and about 1/2 of the total circumference was excised from the cervix. Thus, a rat model of partial cervical defect was established. For two scaffold groups, we implanted recellularized scaffold or decellularized scaffold, respectively, with 6-0 sutures. For the subtotal excision-only group, the uterine cervix with defects was left for spontaneous healing without any scaffolds. And the normal control group underwent a sham laparotomy without any uterine cervix excision. Finally, the abdominal incisions were sutured in double layers by interrupted 3-0 prolene stitches.

### 2.6. Gross Examination and Histochemical Studies

Rats from each group were sacrificed at 7 and 28 days after surgeries for analyses. Gross examination was taken, and the patency of the uterus was tested with methylene blue injection from the ovarian end of the uterus.

The uterine cervix containing the engrafted area was extracted and fabricated into paraffin-embedded sections. Sections were stained with H&E and Masson. The distance from the outermost of the surgical site to the nearest cervical canal lumen was measured as thickness of the cervical surgical site, statistically analyzed and compared for evaluation of cervical repair degree. Masson staining was used to detect cervical fibrosis. The area of blue-stained collagen fibers relative to the total field of view was calculated using ImageJ. Sections were processed for CD31 (Arigo) and Ki-67 (Abcam) in IHC. The neovascular area was measured and expressed as a percentage of CD31 positive area to the total image area. Ki67-positive cells were counted for quantification of cell proliferation.

### 2.7. Evaluation of Fertility

The regenerated uterine cervixes were also assessed for their function in fertility. Rats with recellularized scaffold and decellularized scaffold were mated with fertile males 30 days after surgery. The experiment was terminated about 20 days later to assess graft condition and the number and position of embryos.

### 2.8. Cell Tracing

To trace the implanted recellularized scaffold, ADSCs were labeled with DiO (Beyotime). DiO-labeled ADSCs were used for RC and then implantation. Rats were sacrificed 3 days post implantation, and samples were embedded in Tissue-Tek OCT compound (Sakura, Japan) and frozen for cryosectioning (Leica, Germany). Cell nuclei were stained with DAPI. Images were taken with a Leica SP8 confocal microscope.

### 2.9. Statistics

All statistical analyses were performed using SPSS. Data were shown as the mean ± sd. Student's *t*-test was used for intergroup comparison; one-way ANOVA was used for multiple comparison. *P* < 0.05 was considered statistically significant.

## 3. Results

### 3.1. Evaluation of Decellularized Human Cervix

#### 3.1.1. Histological Evaluation

**Figure 2:**
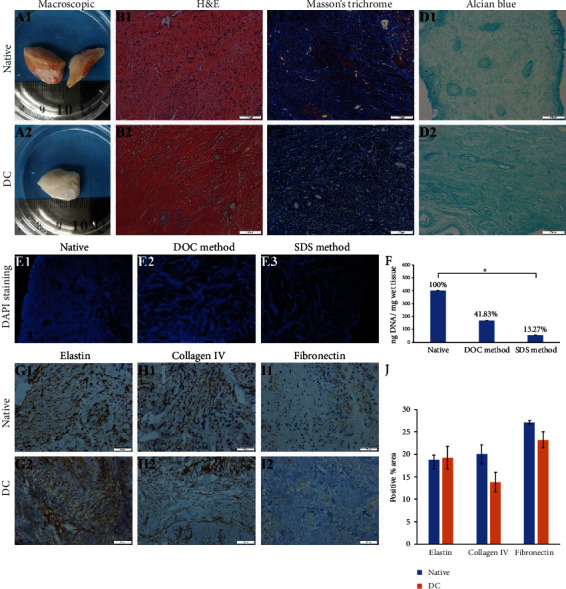
Evaluation of decellularized human cervix. Macroscopic results: the whitening of the tissue was considered as a visual sign of decellularization (a). Representative microscopic images of native human cervical tissue and decellularized scaffolds from SDS method (b–d). Cellular components were stained by H&E (b). ECM components were stained with Masson's trichrome staining (c) and Alcian blue staining (d). Scale bars represent 100 *μ*m for H&E and 200 *μ*m for others. DAPI staining in native tissue (E1), decellularized scaffold from DOC method (E2), and SDS method (E3). Cell nuclei in the native tissue showed a typical round-shape based on DAPI staining. While fluorescence from two methods decreased significantly, DNA was isolated and quantified from native tissue and decellularized scaffolds from two methods (f). Data are shown as mean ± standard deviation. ^∗^*P* < 0.05 indicates statistically significant difference between native tissue and decellularized scaffold from SDS method. IHC staining ECM components including elastin, collagen IV, and fibronectin in native tissue and decellularized scaffold from SDS method are shown (g-i). Scale bars represent 50 *μ*m. No significant change was shown in elastin, collagen IV, and fibronectin after DC (j).

After DC, the samples became whitened and transparent upon visual inspection (Figure 2(a)). H&E staining demonstrated the absence of nuclear components after SDS method (Figure 2(b)). Masson's trichrome staining revealed similar abundant collagen fibers with maintained arrangement after and before DC. The red staining of the cytoplasm was completely removed, while the blue collagen fibers remained (Figure 2(c)). Alcian blue staining showed notable preservation of sulphated glycosaminoglycan (GAG) proteins (Figure 2(d)).

#### 3.1.2. DNA Assessments

DC efficacy was confirmed by DAPI staining and DNA quantification. No obvious blue fluorescence of DAPI-stained cell nuclei was observed after SDS method (Figure 2 (E3)). DNA quantification showed that compared with the native tissues (401.65 ng DNA/mg wet tissue), the remaining DNA of decellularized scaffold from both methods decreased (*P* < 0.05). However, DNA content of SDS method (53.28 ng DNA/mg wet tissue) was significantly lower than that of DOC method (168.03 ng DNA/mg wet tissue) (Figure 2(f)). Therefore, decellularized scaffold from SDS method was selected for IHC and further RC.

#### 3.1.3. Immunohistochemistry

IHC results indicated the presence of major ECM components before and after DC, associated with cell adhesion, cell matrix, and cytoskeletal proteins (Figure 2(j)). Elastin remained abundant before and after DC, giving tissue elasticity and resistance to tension. Collagen IV, the main component of the vascular basement membrane, was uniformly distributed in the decellularized scaffold. And no significant change was shown in fibronectin after DC, a kind of component playing a central role in cell adhesion and regulates cell polarity, differentiation, and growth.

### 3.2. Recellularization of Decellularized Human Cervix

#### 3.2.1. ADSCs

**Figure 3:**
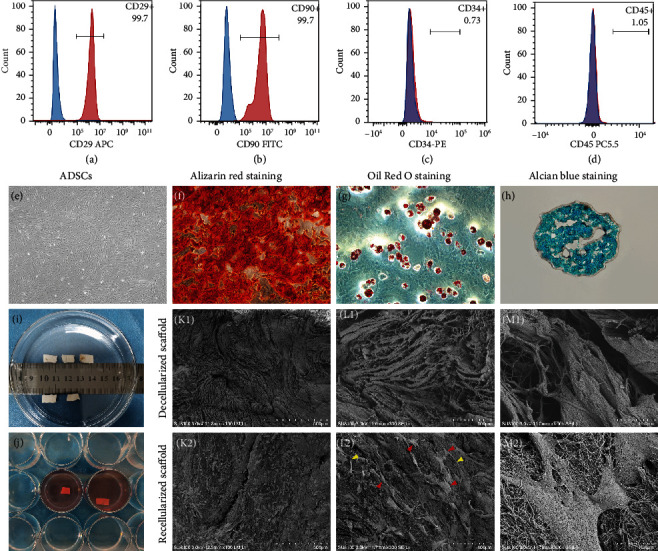
Evaluation of ADSCs and recellularized scaffold. ADSCs were successfully isolated from rat adipose tissues. ADSCs highly expressed CD29 and CD90 but hardly expressed CD34 and CD45 detected by flow cytometry (a–d). ADSCs displayed fibroblast-like spindle shape (e, 4x). Calcium deposit was stained with Alizarin Red (f, 10x). Adipogenic vesicles were observed by Oil Red O staining (g, 20x). Glycosaminoglycan (GAG) in the cartilage matrix nodule was stained with Alcian blue (h, 20x). The decellularized scaffolds were dried by an experimental vacuum freeze dryer and sliced to pieces (i) and then recellularized (j). The structures of decellularized scaffold (K1-M1) and recellularized scaffold (K2-M2) were observed via scanning electron microscopy at different magnifications. After RC, ADSCs filled the gaps shown in the decellularized scaffold while displaying out-grown morphology. Red arrows indicate ADSCs. Yellow arrows indicate cellular extension and a network between ADSCs. Scale bars represent 500 *μ*m (k), 100 *μ*m (l), and 10 *μ*m (m), respectively.

The cells exhibited spindle-shaped morphology and attached well to the culture dish (Figure 3(e)). Flow cytometric analysis revealed that the cells were positive for CD29 (99.7%) and CD90 (99.7%) and negative for CD34 (0.73%) and CD45 (1.05%) (Figures 3(a)–3(d)). And they were successfully differentiated into osteogenic, adipogenic, and chondrogenic lineages in vitro under suitable differentiation conditions (Figures 3(f)–3(h)). These results indicated that the cells we cultured were ADSCs with multilineage differentiation potential.

#### 3.2.2. Recellularization and SEM

ADSCs were seeded carefully to the decellularized scaffold (Figure 3(j)). SEM images indicated a large number of collagen fibers orderly distributed in the decellularized scaffold with some empty pores which were considered as vacancies derived from cell removal (Figure 3 (K1-M1)). These findings could be interpreted as the preservation of the ECM microstructures after DC. ADSCs attached and distributed evenly on the recellularized scaffold, with cellular extension and a network between cells observed (Figure 3 (K2-M2)). These results indicated the scaffold provided a favorable environment for seeded cells, which can also be confirmed in cell tracing.

### 3.3. Reconstruction of Partial Uterine Cervical Defect in Rats

**Figure 4:**
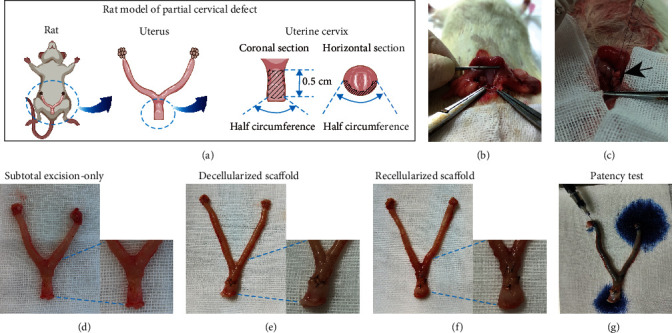
Surgical processes and gross examination of reconstruction of partial uterine cervical defect in rat. The schematic diagram of establishing mouse model of partial cervical injury (a). Surgical processes for two scaffold groups were shown: after exposure of uterine cervix (b), a tissue segment was excised from the cervix, then scaffold was implanted, respectively, with 6-0 sutures (c). The black arrow indicates the implantation site. Gross observation of rat uterus from the subtotal excision-only group (d), decellularized scaffold group (e), and recellularized scaffold group (f) was shown, respectively. Uterine patency test showed that the solution flowed freely out of the cervical end of the uterus, with the recellularized scaffold group as an example (g).

All rats survived during the surgeries. The schematic diagram of establishing rat model and some of the classic surgery photos of implantation are illustrated in Figure 4.

#### 3.3.1. Gross Examination

In the scaffold groups, the implantation sites could be located by sutures, and no adhesion was observed. 28 days after surgery, recellularized scaffold and decellularized scaffold both showed integration into adjacent tissue. The subtotal excision-only group showed fibrotic scars and mild atrophy on the surgical site. Uterine patency test showed that the solution flowed freely from the cervical end of the uterus in all groups. The uterine patency of both scaffold groups was also confirmed by fertility tests.

#### 3.3.2. Histological Evaluation

**Figure 5:**
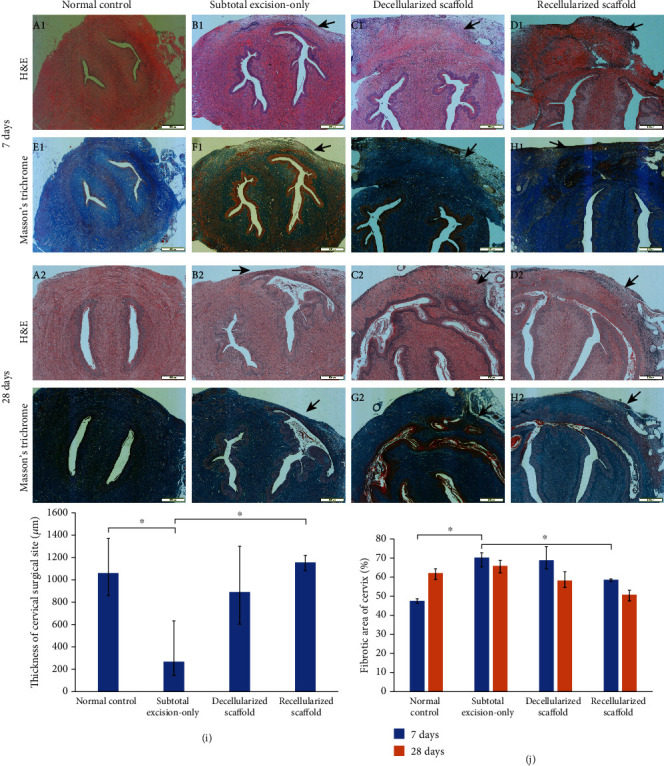
Histological evaluation of reconstruction of partial uterine cervical defect in rat. Histological images of cross-sections of rat uterine cervix from the subtotal excision-only group, decellularized scaffold group, and recellularized scaffold group were shown at 7 days (A1-H1) and 28 days (A2-H2) postsurgery. (a–d) are for H&E staining, and (e–h) are for Masson's trichrome staining. Black arrows indicate the surgical sites of each group. Scale bars represent 500 *μ*m. Statistical analysis of the thickness of cervical surgical site (i) finds that the cervical surgical site in both scaffold groups was thicker than the subtotal excision-only group and was similar to the normal control group. Masson staining illustrated collagen fibers as blue fibers and was used to detect cervical fibrosis (j). The proportion of fibrotic area of the recellularized scaffold group and normal control group was lower than that of the subtotal excision-only group. There were no significant differences in fibrosis between 7 days and 28 days after surgery in all groups. Data are presented as mean ± SEM. ^∗^*P* < 0.05.

Seven days after surgery, the scaffold groups showed high cell density among the fibers and slightly disordered fiber arrangement. Compared with the decellularized scaffold group, the recellularized scaffold group had more obvious neovascularization and cellularization. 28 days after surgery, the implants fused better with adjacent tissues, and the muscle bundles were arranged more orderly in scaffold groups. However, the subtotal excision-only group showed scar formation and deficiency of native cells. The thickness of cervical surgical sites in the recellularized scaffold group and decellularized scaffold group (1156.79 ± 55.64 *μ*m and 891.81 ± 296.59 *μ*m) was similar to that of the normal control group (1061.34 ± 222.23 *μ*m). And the recellularized scaffold group was significantly higher than that of the subtotal excision-only group (169.57 ± 268.53 *μ*m) (Figure 5(i)). Masson's trichrome staining showed that the proportion of fibrotic area of the recellularized scaffold group was lower than that of the subtotal excision-only group (Figure 5(j)).

#### 3.3.3. Immunohistochemistry

**Figure 6:**
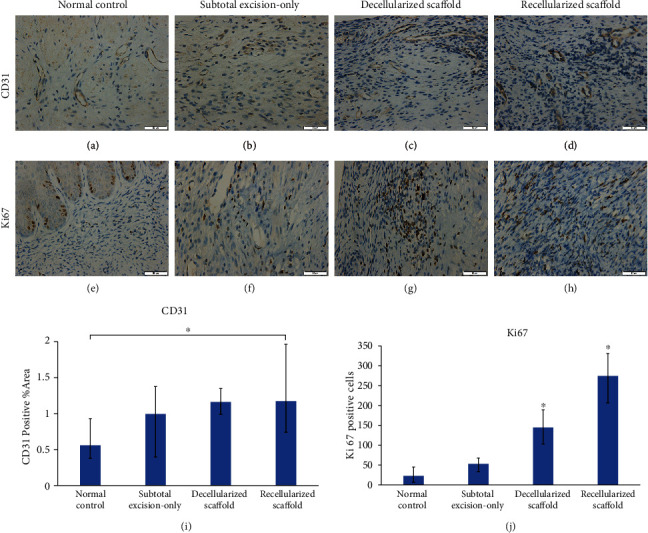
Immunohistochemistry of reconstruction of partial uterine cervical defect in rat. Immunostaining of CD31 (a–d) and Ki67 (e–h), respectively, in normal control (a, e), subtotal excision-only (b, d), decellularized scaffold (c, e), and recellularized scaffold (d, h) groups. Scale bars represent 50 *μ*m. The CD31 positive neovascular area of the recellularized scaffold group was significantly higher than that of the normal control group (i). The normal control group was the lowest in Ki67-positive expression with a higher concentration of positive-stained cells in the luminal epithelium. The scaffold groups were all significantly higher than the other two groups, with the recellularized scaffold group being the highest. ^∗^*P* < 0.05.

CD31 is a specific endothelial marker used to detect angiogenesis. IHC showed diffused positive CD31 staining in both scaffold groups. And neovascular area of the recellularized scaffold group was significantly higher than that of the normal control group, which suggested ideal angiogenesis of the implants (Figure 6(i)). In the process of cervical tissue repair, active cell proliferation is beneficial to promote tissue repair. Ki67 indicated the proliferation capacity of implants. The normal control group showed weak positive Ki67 while both scaffold groups revealed significantly stronger positive reaction for Ki67 staining in all parts of surgical site than other groups, which confirmed the regenerative capacity of implants (Figure 6(j)).

#### 3.3.4. Evaluation of Fertility

**Figure 7:**
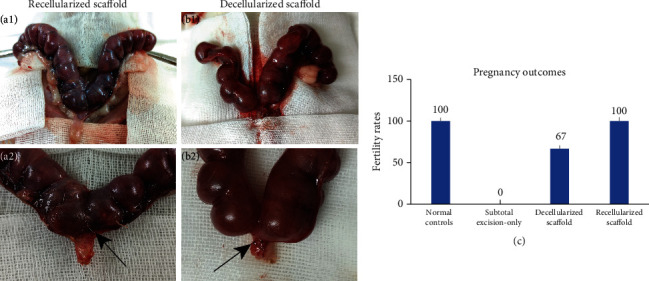
Pregnancy outcomes of fertility test. Positive pregnancy outcomes in recellularized scaffold (a1, a2) and decellularized scaffold (b1, b2) groups. The implantation sites can be indicated by visible sutures. Black arrows indicate the surgical sites of the cervixes. The figure shows the fertility rate of four groups (c).

20 days after pregnancy experiment, all rats were sacrificed. Atrophy and structural deformation of the wounded cervix were observed in the subtotal excision-only group, while no pregnancy was observed. Pregnancy failures were suspected to be due to cervical stenosis or obstruction caused by excision surgery. In the recellularized scaffold group, all rats were pregnant with similar number of embryos as normal (Figure 7). One rat delivered healthy pups, which proved the repaired cervix can withstand expansion during pregnancy. All but one rat were pregnant in the decellularized scaffold group, and hyperemia was observed in the nonpregnant rat. Embryos existed in both sides of the uteruses of pregnant rats. These results implied that the recellularized scaffold could repair the cervix and promote fertility recovery.

### 3.4. Cell Tracing In Vivo

**Figure 8:**
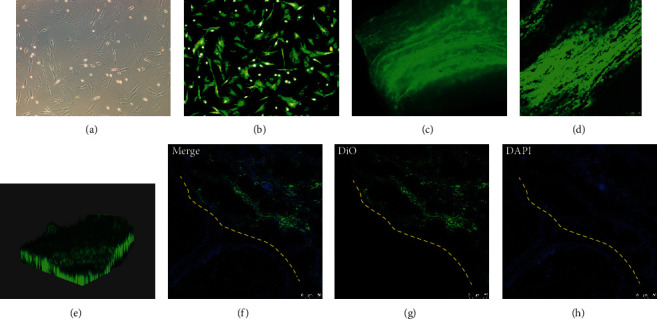
Cell tracing in vivo results. ADSCs were labeled with DiO (green) (a, b, 10x). Three days after RC with DiO-labeled ADSCs, microscopic images (c) and confocal images showed diffuse green fluorescence throughout the recellularized scaffold. By serial confocal imaging of the scaffold, herein showed fluorescent image of one random transverse section of the scaffold (d) and confocal 3D images (e). Scaffold with DiO-labeled ADSCs was implanted to the rat model, and microphotographs of cross-sections of the rat uterine cervix were taken 3 days after surgery. Merge (f), DAPI (h), and DiO (g) images of fluorescence signals are shown, respectively. Nuclei of DiO-labeled transplanted donor cells and host cells were all stained blue with DAPI and distributed in all parts of the tissue, while green fluorescence with DiO of donor cells only existed in the implant site. Yellow dashed lines represent the border between graft and adjacent original tissue. Scale bars represent 50 *μ*m.

In this study, DiO-labeled ADSCs showed typically spindle-shaped morphology and exhibited stable expression of green fluorescence in vitro (Figure 8(b)). Microscopic observation revealed that fluorescence intensity was not markedly decreased within a week of culture. After RC, microscopic images (Figure 8(c)) showed diffuse green fluorescence throughout the scaffold, which proved that the cells migrated into the scaffold and proliferated. To better visualize the state of the cells inside the scaffold, serial confocal imaging began at the surface of the scaffold and extended to full depth.  Figure 8(d) shows a fluorescent image of one random transverse section of the scaffold, and the distributions of DiO were imaged by 3-dimensional (3D) confocal microscopy (Figure 8(e)). Three days post implantation, lots of green fluorescence were found notably in the implant site, not in the adjacent original tissue (Figure 8(f)), showing that the donor cells survived and exerted functional effects in vivo.

## 4. Discussion

Cervical aplasia is usually diagnosed in adolescence, since obstruction of the outflow tract results in an anterograde accumulation of the blood flow. Delays in treatment can increase incidence of endometriosis, along with impairment of the normal function of the fallopian tubes and ovaries [22]. However, therapeutic strategy presents a challenge for gynecologists. Tissue engineering for obtaining a functional bioengineered cervix is a promising treatment strategy for cervical aplasia. In our study, we reconstructed the partial cervical defect in a rat model with a decellularized human cervical tissue combined with ADSCs, achieving both anatomical recovery and functional restoration.

Decellularization is an innovative approach to obtaining natural scaffolds, which overcomes the problem of low compatibility of donor organs and need for long-term immunosuppressant therapy by removing cellular antigens. The choice of DC detergents affects the characteristics of the bioengineered construct. Therefore, we aimed to find appropriate protocols that efficiently remove cellular components while preserving the native ECM. Our first protocol was based on SDS, a strong detergent considered as the best choice for sheep uterus decellularization in an earlier study [23]. The other protocol chose DOC, probably superior to SDS as milder detergent for all tissue types except the heart in the interest of preserving the collagen and elastin structure [24]. Concentrations were referenced from previous animal studies, and the timing was adjusted through the decellularization processes to better accommodate human uterine tissue. DNA assessments showed that both protocols could not completely remove DNA. Nonetheless, SDS method could remove DNA up to 87% compared to native. Residual DNA could be associated with insufficient washings, which can be adjusted by reducing the thickness of tissue slices to promote adequate infiltration of tissue by detergent. However, though the residual quantity of DNA may exceed that of previously accepted criteria [17], the significance of this is still in discussion [25]. For example, aortic root scaffolds with much higher DNA contents have shown no tissue rejection after implantation [26]. Importantly, our in vivo implantation experiment did not show any adverse immunological response to the decellularized scaffold, which suggested that the impact of remaining DNA might not be absolutely crucial. Furthermore, methods to enhance DNA removal such as higher concentrations of reagents or longer treatment time may damage or denature ECM components which decrease tissue regeneration properties.

ECM is a highly dynamic three-dimensional noncellular polymer network composed of collagen, glycosaminoglycan, elastin, fibronectin, laminin, etc. Previous studies demonstrated that ECM not only provides structural support for cell embedding but also participates in the regulation of many cellular processes including growth, migration, differentiation, survival, and homeostasis [27–29]. Moreover, an innovative study by Campo et al. showed that in vitro embryo development was supported by the acellular ECM from synchronous endometrial coating, which achieved similar results to the gold standard that only uses serum-containing media [30]. This reveals that DC preserves the intraspecificity of developing hormonally stimulated tissues. The tissues used in our study were all from hysterectomy for benign lesions, with small sample size and menstrual cycle were not taken into account. Subsequent studies are expected to compare the properties of decellularized ECM including hormone levels, in premenopausal and postmenopausal uterine tissues to optimize the composition of the engineered cervix. In addition, although decellularization can theoretically remove cellular components, ECM is one important constituent of the tumor microenvironment [31, 32]. Thus, we do not encourage the selection of postoperative tissue from patients with malignant tumors, to reduce the risk of a new onset cancer. Herein, we confirmed the preservation of major components of ECM by histochemical studies and SEM. And the possible mechanical properties of ECM were confirmed by the presence of native collagen fibers visualized with Masson and Alcian blue staining [15]. Thus, it is evident that ECM was preserved to the maximum extent possible, providing a viable scaffold for future recellularization (RC).

In the following RC, the preservation of ECM provides a satisfactory environment for cell attachment. SEM suggested that our DC protocol generated a porous acellular biomaterial that featured a denser fiber structure. Importantly, the scaffold areas presented adequate cell population and distribution, indicating cells could survive properly within the scaffold, showing RC was efficient after the injection of ADSCs. The successful follow-up of fluorescence signal after implantation in vivo also confirmed that the seeded cells grew and proliferated smoothly, which further verified the biocompatibility of the scaffolds. For further studies, we found it critical to analyze the RC process and results prior to implantation, since a properly high RC efficiency will facilitate further cell repopulation in vivo. Then, we further explored the repair function of recellularized scaffold for cervical defect in a rat model. After 28 days follow-up, we observed a complete cervical repair in recellularized scaffold, similar with normal control. Meanwhile, it could produce better thickness of cervical surgical site and less fibrotic area compared with pure decellularized scaffold. It is known that vasculature is an essential component of almost any tissue or organ, and therefore, angiogenesis is critical to the success of bioengineered implants. Some studies suggested the addition of exogenous growth factors or the use of engineered [24] and pharmacologically modified ADSCs [33] to promote vascularization. IHC results confirmed a more positive vascularization marker CD31 and proliferation marker Ki67 in recellularized scaffold. The mechanism by which ADSCs participate in tissue repair is unclear, but possibilities include direct differentiation of ADSCs, release of cytokines or growth factors, and enhanced cellular recruitment through paracrine/autocrine effects. In a study of enhancing wound healing in diabetes, ADSC injection treatment stimulated neoangiogenesis and increased suppression of the topical tissue inflammatory response through paracrine and autocrine mechanisms [34]. In addition, Lu et al. have found that ADSCs can enhance the blood supply of random pattern skin flaps, and the mechanism may be related to the direct differentiation of ADSCs into endothelial cells and the indirect effect of angiogenic growth factors released by ADSCs [35]. However, our study did not show that the cells used herein presented any signs of differentiating into uterus-specific cell types. A heterogeneous cell mix of cell types or specific homogenous cell populations added in sequence may improve the outcomes according to a study [36]. Thus, it may be appropriate to evaluate more uterus-like cells in future experiments for the repair of cervical defect.

Finally, based on positive pregnancy results in scaffold groups, our constructs functioned well and gave proper support during pregnancy. Considering the special anatomical location, the smooth passage of the cervix is a prerequisite for successful mating. It is apparent that expansion of the gravid uterus and extension of cervix exist in the stage of pregnancy, which confirmed the good biomechanical properties of tissue engineering repaired cervix. It is undeniable that decellularized scaffold also showed certain cervical repair ability in our evaluations, which is considered relying on migration and repopulation of autologous cells of the transplant recipient. Earlier studies suggested that even without RC [18], uterine tissue engineering strategies are effective in repair for severe defect on the uterus. However, our experiment was aimed at cervical tissues, with the expectation to be applied to larger and more complete organ/tissue subsequently, so as to realize cervical reconstruction for patients with the absolute absence of any cervical tissue or severely defected cervical tissue in the future. In this aspect, pure decellularized material itself may not be dominant as it relies solely on migration of autologous cells, and it is more likely to lead to thrombosis. We highlight the benefits of decellularized scaffold to create an in vitro supportive environment for supplementing in vivo tissue regeneration. Appropriate recellularization contributes to specific differentiation of cells and formation of functional vascular network. Therefore, we prefer to choose recellularized cervical scaffold for further research and application.

In conclusion, tissue engineering cervical tissue has been successfully constructed by decellularized scaffold combined with ADSCs, closely resembling native cervical tissue in morphology and function in rat model. This study provides experimental basis for the successful application of cervical tissue engineering in clinical practice in the future.

## Data Availability

The data used to support the findings of this study are included within the article.
